# 1-(2-Methyl­imidazo[1,2-*a*]pyridin-3-yl)-3,3-bis­(methyl­sulfan­yl)prop-2-enone monohydrate

**DOI:** 10.1107/S1600536809023514

**Published:** 2009-06-27

**Authors:** Yvon Bibila Mayaya Bisseyou, Drissa Sissouma, Severin D. Goulizan Bi, Mahama Ouattara, R. C. A. Yao-Kakou

**Affiliations:** a Laboratoire de Cristallographie et Physique Moléculaire, UFR SSMT, Université de Cocody, 22 BP 582 Abidjan 22, Côte d’Ivoire; b Laboratoire de Chimie Organique Structurale, UFR SSMT, Université de Cocody, 22 BP 582 Abidjan 22, Côte d’Ivoire; c Laboratoire de Chimie Thérapeutique et Synthèse de Médicaments, UFR Sciences Pharmaceutiques, Université de Cocody, 01 BP V 34 Abidjan 01, Côte d’Ivoire

## Abstract

The title compound, C_13_H_14_N_2_OS_2_·H_2_O, appears in the form of bimolecular aggregate in which mol­ecular components are linked by O—H⋯N hydrogen bonding. The nine-membered imidazo[1,2-*a*]pyridine system is almost planar, with a mean deviation of 0.026 (1) Å. An intra­molecular C—H⋯O hydrogen bond forms within the imidazo[1,2-*a*]pyridine system. The crystal packing is consolidated by O—H⋯O and C—H⋯O hydrogen bonds, forming a supra­molecular structure consisting of perpendicular infinite mol­ecular chains running along the *a* and *c* axes.

## Related literature

For related structures, see: Bibila Mayaya Bisseyou *et al.* (2007 ▶, 2009 ▶); Duan *et al.*(2006 ▶). For hydrogen-bond motifs, see: Bernstein *et al.* (1995 ▶).
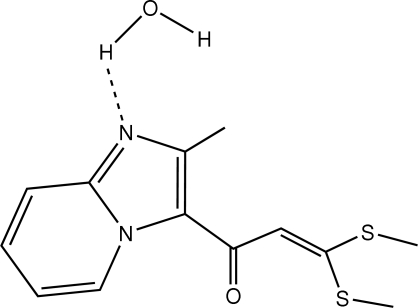


## Experimental

### 

#### Crystal data

C_13_H_14_N_2_OS_2_·H_2_O
*M* *_r_* = 296.41
Orthorhombic, 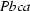

*a* = 5.1405 (1) Å
*b* = 17.7653 (3) Å
*c* = 31.3919 (6) Å
*V* = 2866.79 (9) Å^3^
*Z* = 8
Mo *K*α radiation
μ = 0.37 mm^−1^
*T* = 295 K
0.25 × 0.15 × 0.15 mm

#### Data collection

Nonius KappaCCD diffractometer
Absorption correction: multi-scan *DENZO*/*SCALEPACK* (Otwinowski & Minor, 1997 ▶) *T*_min_ = 0.90, *T*_max_ = 0.95
11253 measured reflections
4202 independent reflections
2344 reflections with *I* > 2σ(*I*)
*R*_int_ = 0.05

#### Refinement

*R*[*F* ^2^ > 2σ(*F* ^2^)] = 0.047
*wR*(*F* ^2^) = 0.097
*S* = 0.99
4202 reflections
172 parameters
H-atom parameters constrained
Δρ_max_ = 0.18 e Å^−3^
Δρ_min_ = −0.25 e Å^−3^

### 

Data collection: *COLLECT* (Nonius, 1997 ▶); cell refinement: *DENZO*/*SCALEPACK* (Otwinowski & Minor, 1997 ▶); data reduction: *DENZO*/*SCALEPACK*; program(s) used to solve structure: *SIR92* (Altomare *et al.*, 1994 ▶); program(s) used to refine structure: *CRYSTALS* (Betteridge *et al.*, 2003 ▶); molecular graphics: *ORTEP-3* (Farrugia, 1997 ▶) and *PLATON* (Spek, 2009 ▶); software used to prepare material for publication: *CRYSTALS*.

## Supplementary Material

Crystal structure: contains datablocks I, global. DOI: 10.1107/S1600536809023514/bq2145sup1.cif

Structure factors: contains datablocks I. DOI: 10.1107/S1600536809023514/bq2145Isup2.hkl

Additional supplementary materials:  crystallographic information; 3D view; checkCIF report

## Figures and Tables

**Table 1:** Hydrogen-bond geometry (Å, °)

*D*—H⋯*A*	*D*—H	H⋯*A*	*D*⋯*A*	*D*—H⋯*A*
C4—H4⋯O1	0.95	2.23	2.840 (4)	121
C3—H3⋯O1^i^	0.93	2.45	3.242 (4)	143
O2*w*—H22*w*⋯O2*w*^ii^	0.82	2.05	2.862 (4)	168
O2*w*—H21*w*⋯N2	0.82	2.06	2.849 (4)	164

Symmetry codes: (i) 
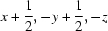
; (ii) 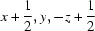.

**Fig. 1.**
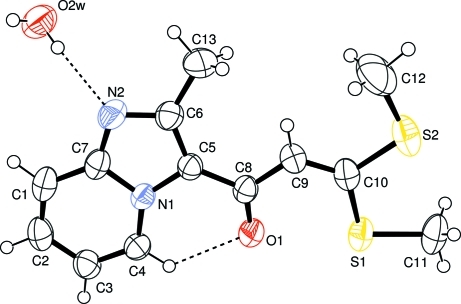
The molecular structure of the title compound and the atomic numbering scheme. Displacement ellipsoids are drawn at the 50% probability level. H atoms are shown as spheres of arbitrary radius. Dashed lines indicate hydrogen bonds.

**Fig. 2.**
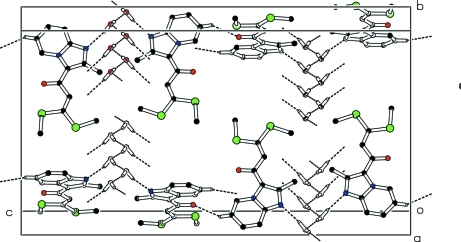
Crystal packing of the title compound, viewed down the a axis, showing the both perpendicular infinite chain types. The first one is established by bimolecular aggregates along c axis; the second one is parallel to a axis and constituted only by water molecules in zigzag fashion. H atoms not involved in hydrogen bonds have been omitted for clarity. Dashed lines indicate hydrogen bonds.

## supplementary crystallographic information

### Comment

As part of continuing work on heterocyclic compounds biologically active, we have synthesized new derivative in order to explore new potential activities or to improve known properties of this compound class. Asymmetric unit of title compound which crystallize as the monohydrate is consist of two independent molecular components connected between them by an O—H···N hydrogen bond forming thus one bimolecular aggregate (Fig. 1). In the imidazo[1,2-*a*]pyridine component, the values of bond lengths and angles of nine-membered imidazopyridine ring are similar to those found in previous studies (Bibila Mayaya Bisseyou *et al.*, 2007, 2009, Duan *et al.*, 2006). Imidazopyridine ring, essentially planar with mean deviation of 0.026 (1)Å, makes dihedral angles of 6.11 (23)° and 9.79 (15)° with P1(C9/C10/S1/C11) and P2(C9/C10/S2/C12) mean planes, respectively. Besides, we also note within of the same component, the presence of C—H···O intra-molecular hydrogen bond (Table 1) generating an S(6) motif (Bernstein *et al.*, 1995). The three-dimensional crystal packing is consolidated by inter-bimolecular aggregate hydrogen bonds (Fig. 2). Indeed, each bimolecular unit is linked to three others *via* O—H···O and C—H···O hydrogen bonds (Table 1) forming thus a supra-molecular structure consisted of to two perpendicular infinite chain types: the first one is established by bimolecular aggregates along *c* axis; the second one is parallel to *a* axis and constituted only by water molecules in zigzag fashion.

### Experimental

1-(2-methylimidazo[1,2-*a*]pyridine-3-yl) acetyl (1 g, 6.2 mmol) was dissolved in distilled dimethyl sulfoxide (15 ml), and the carbon disulfur (0.41 ml, 6.8 mmol) was added. After cooling of the mixture at 0°C, sodium hydrure (0.36 g, 15.4 mmol) was added. After stirring for 30 minutes at 0°C, the mixture was stirred at ambient temperature during 4 h. Solution was then cooled at 0°C and methyl iodide (2.5 molar equivalents) was added dropwise. The resulting mixture was then left under stirring during 24 h then poured into 50 ml ice-cold water. The precipitate was filtered and recrystallized from mixture of water-dioxane (2:1) to obtain orange single crystals of title compound (1.31 g; yield, 80%, m.p.: 430 K)

### Refinement

The H atoms were all located in a difference of Fourier map. They were all initially refined with soft restraints on the bond lengths and angles to regularize their geometry (C—H in the range 0.95–0.97Å, O—H=0.82Å and *U*_iso_(H) in the range 1.2–1.5 times *U*_eq_ of the parent atom), after which their positions were refined with riding constraints.

### Crystal data

**Table:**

C_13_H_14_N_2_OS_2_·H_2_O	*D*_x_ = 1.373 Mg m^−^^3^
*M**_r_* = 296.41	Melting point: 430 K
Orthorhombic, *P**b**c**a*	Mo *K*α radiation, λ = 0.71073 Å
Hall symbol: -P 2ac 2ab	Cell parameters from 11253 reflections
*a* = 5.1405 (1) Å	θ = 4–30°
*b* = 17.7653 (3) Å	µ = 0.37 mm^−^^1^
*c* = 31.3919 (6) Å	*T* = 295 K
*V* = 2866.79 (9) Å^3^	Prism, orange
*Z* = 8	0.25 × 0.15 × 0.15 mm
*F*(000) = 1248	

### Data collection

**Table:**

Nonius KappaCCD diffractometer	2344 reflections with *I* > 3σ(*I*)
graphite	*R*_int_ = 0.05
φ scans	θ_max_ = 30.5°, θ_min_ = 1.3°
Absorption correction: multi-scan *DENZO*/*SCALEPACK* (Otwinowski & Minor, 1997)	*h* = 0→7
*T*_min_ = 0.90, *T*_max_ = 0.95	*k* = 0→25
11253 measured reflections	*l* = 0→43
4202 independent reflections	

### Refinement

**Table:**

Refinement on *F*^2^	Primary atom site location: structure-invariant direct methods
Least-squares matrix: full	Hydrogen site location: inferred from neighbouring sites
*R*[*F*^2^ > 2σ(*F*^2^)] = 0.047	H-atom parameters constrained
*wR*(*F*^2^) = 0.097	*w* = 1/[σ^2^(*F*^2^) + (0.04*P*)^2^ + 3.2*P*], where *P* = [max(*F*_o_^2^,0) + 2*F*_c_^2^]/3
*S* = 0.99	(Δ/σ)_max_ = 0.0002
2066 reflections	Δρ_max_ = 0.18 e Å^−^^3^
172 parameters	Δρ_min_ = −0.25 e Å^−^^3^
0 restraints	

### Fractional atomic coordinates and isotropic or equivalent isotropic displacement parameters (Å^2^)

**Table:**

	*x*	*y*	*z*	*U*_iso_*/*U*_eq_	
S1	0.01264 (14)	0.40723 (4)	0.05322 (3)	0.0552	
S2	−0.11568 (16)	0.46016 (4)	0.14025 (3)	0.0696	
O1	0.3999 (4)	0.30577 (10)	0.05889 (6)	0.0542	
N1	0.7820 (4)	0.21236 (11)	0.09663 (7)	0.0394	
O2w	0.9388 (4)	0.07731 (12)	0.22997 (7)	0.0739	
N2	0.8459 (5)	0.18741 (13)	0.16574 (8)	0.0559	
C4	0.8270 (5)	0.20481 (13)	0.05354 (9)	0.0464	
C7	0.9265 (5)	0.17257 (14)	0.12603 (9)	0.0474	
C9	0.2551 (5)	0.35366 (14)	0.12396 (9)	0.0472	
C5	0.6013 (5)	0.25573 (13)	0.11962 (8)	0.0410	
C3	1.0207 (6)	0.15826 (14)	0.04040 (10)	0.0525	
C2	1.1724 (6)	0.11813 (15)	0.06988 (11)	0.0573	
C1	1.1255 (6)	0.12480 (15)	0.11196 (11)	0.0561	
C6	0.6478 (5)	0.23742 (16)	0.16209 (9)	0.0494	
C10	0.0715 (5)	0.40096 (14)	0.10800 (9)	0.0473	
C8	0.4160 (5)	0.30513 (13)	0.09821 (9)	0.0417	
C11	−0.2533 (6)	0.47334 (16)	0.04946 (12)	0.0704	
C13	0.5083 (7)	0.2619 (2)	0.20158 (10)	0.0723	
C12	0.0018 (9)	0.4409 (2)	0.19285 (13)	0.0992	
H2	1.3072	0.0877	0.0601	0.0687*	
H4	0.7197	0.2326	0.0346	0.0557*	
H9	0.2801	0.3530	0.1530	0.0562*	
H1	1.2232	0.0990	0.1323	0.0665*	
H113	−0.2078	0.5201	0.0623	0.1052*	
H112	−0.2875	0.4811	0.0199	0.1058*	
H111	−0.4050	0.4535	0.0630	0.1059*	
H3	1.0530	0.1538	0.0113	0.0637*	
H132	0.5316	0.3152	0.2061	0.1075*	
H133	0.3256	0.2531	0.1989	0.1079*	
H131	0.5739	0.2357	0.2257	0.1077*	
H22w	1.0875	0.0824	0.2392	0.1108*	
H123	−0.0206	0.3876	0.1991	0.1488*	
H21w	0.9353	0.1136	0.2140	0.1109*	
H122	0.1833	0.4544	0.1939	0.1486*	
H121	−0.0960	0.4706	0.2124	0.1492*	

### Atomic displacement parameters (Å^2^)

**Table:**

	*U*^11^	*U*^22^	*U*^33^	*U*^12^	*U*^13^	*U*^23^
S1	0.0444 (4)	0.0482 (4)	0.0731 (5)	0.0067 (3)	−0.0097 (4)	0.0037 (4)
S2	0.0549 (5)	0.0552 (4)	0.0988 (7)	0.0090 (4)	0.0082 (4)	−0.0195 (4)
O1	0.0542 (11)	0.0608 (12)	0.0477 (13)	0.0151 (9)	−0.0031 (10)	0.0060 (9)
N1	0.0375 (11)	0.0360 (10)	0.0448 (12)	−0.0011 (9)	−0.0041 (10)	0.0044 (9)
O2w	0.0700 (15)	0.0794 (15)	0.0723 (16)	−0.0039 (12)	−0.0076 (12)	0.0241 (12)
N2	0.0551 (15)	0.0623 (15)	0.0501 (15)	0.0054 (12)	−0.0097 (12)	0.0093 (12)
C4	0.0497 (16)	0.0397 (13)	0.0496 (17)	0.0002 (11)	0.0008 (12)	0.0037 (12)
C7	0.0416 (15)	0.0434 (13)	0.0572 (18)	0.0000 (11)	−0.0097 (13)	0.0060 (13)
C9	0.0414 (14)	0.0488 (14)	0.0515 (16)	0.0037 (12)	0.0006 (13)	−0.0051 (12)
C5	0.0362 (13)	0.0405 (13)	0.0464 (16)	−0.0010 (11)	−0.0001 (12)	0.0028 (11)
C3	0.0550 (17)	0.0431 (14)	0.0594 (18)	0.0028 (13)	0.0065 (14)	−0.0017 (12)
C2	0.0493 (17)	0.0444 (15)	0.078 (2)	0.0084 (13)	0.0018 (15)	−0.0043 (15)
C1	0.0497 (17)	0.0448 (14)	0.074 (2)	0.0076 (13)	−0.0120 (15)	0.0049 (14)
C6	0.0454 (15)	0.0552 (16)	0.0476 (17)	0.0005 (13)	−0.0047 (13)	0.0023 (13)
C10	0.0356 (14)	0.0381 (13)	0.068 (2)	−0.0033 (11)	0.0012 (12)	−0.0048 (12)
C8	0.0356 (13)	0.0400 (12)	0.0495 (17)	−0.0016 (10)	−0.0039 (12)	0.0026 (12)
C11	0.0485 (17)	0.0517 (17)	0.111 (3)	0.0064 (14)	−0.0165 (19)	0.0092 (18)
C13	0.076 (2)	0.098 (2)	0.0430 (18)	0.015 (2)	−0.0014 (17)	0.0030 (17)
C12	0.112 (3)	0.105 (3)	0.081 (3)	0.021 (3)	0.016 (2)	−0.035 (2)

### Geometric parameters (Å, °)

**Table:**

S1—C10	1.750 (3)	C5—C6	1.393 (4)
S1—C11	1.806 (3)	C5—C8	1.459 (3)
S2—C10	1.748 (3)	C3—C2	1.405 (4)
S2—C12	1.791 (4)	C3—H3	0.930
O1—C8	1.237 (3)	C2—C1	1.348 (4)
N1—C4	1.379 (3)	C2—H2	0.931
N1—C7	1.380 (3)	C1—H1	0.932
N1—C5	1.406 (3)	C6—C13	1.496 (4)
O2w—H22w	0.822	C11—H113	0.952
O2w—H21w	0.817	C11—H112	0.954
N2—C7	1.340 (4)	C11—H111	0.955
N2—C6	1.356 (3)	C13—H132	0.966
C4—C3	1.358 (4)	C13—H133	0.956
C4—H4	0.948	C13—H131	0.951
C7—C1	1.401 (4)	C12—H123	0.974
C9—C10	1.359 (3)	C12—H122	0.964
C9—C8	1.443 (4)	C12—H121	0.953
C9—H9	0.922		
			
C10—S1—C11	103.68 (15)	C5—C6—N2	111.3 (2)
C10—S2—C12	103.50 (16)	C5—C6—C13	130.0 (3)
C4—N1—C7	121.1 (2)	N2—C6—C13	118.7 (3)
C4—N1—C5	131.9 (2)	S1—C10—S2	115.83 (15)
C7—N1—C5	107.0 (2)	S1—C10—C9	121.4 (2)
H22w—O2w—H21w	98.6	S2—C10—C9	122.7 (2)
C7—N2—C6	106.4 (2)	C5—C8—C9	118.4 (2)
N1—C4—C3	118.7 (3)	C5—C8—O1	120.6 (2)
N1—C4—H4	117.7	C9—C8—O1	121.0 (2)
C3—C4—H4	123.6	S1—C11—H113	110.7
N1—C7—N2	110.8 (2)	S1—C11—H112	107.3
N1—C7—C1	119.5 (3)	H113—C11—H112	109.3
N2—C7—C1	129.7 (3)	S1—C11—H111	110.4
C10—C9—C8	124.1 (3)	H113—C11—H111	109.6
C10—C9—H9	118.0	H112—C11—H111	109.5
C8—C9—H9	117.8	C6—C13—H132	110.3
N1—C5—C6	104.5 (2)	C6—C13—H133	110.5
N1—C5—C8	121.6 (2)	H132—C13—H133	107.1
C6—C5—C8	133.9 (2)	C6—C13—H131	110.4
C4—C3—C2	121.1 (3)	H132—C13—H131	108.6
C4—C3—H3	118.7	H133—C13—H131	109.9
C2—C3—H3	120.3	S2—C12—H123	109.3
C3—C2—C1	120.1 (3)	S2—C12—H122	108.2
C3—C2—H2	119.3	H123—C12—H122	110.5
C1—C2—H2	120.5	S2—C12—H121	108.0
C7—C1—C2	119.5 (3)	H123—C12—H121	110.3
C7—C1—H1	118.4	H122—C12—H121	110.5
C2—C1—H1	122.0		

### Hydrogen-bond geometry (Å, °)

**Table:**

*D*—H···*A*	*D*—H	H···*A*	*D*···*A*	*D*—H···*A*
C4—H4···O1	0.95	2.23	2.840 (4)	121
C3—H3···O1^i^	0.93	2.45	3.242 (4)	143
O2w—H22w···O2w^ii^	0.82	2.05	2.862 (4)	168
O2w—H21w···N2	0.82	2.06	2.849 (4)	164

Symmetry codes: (i) *x*+1/2, −*y*+1/2, −*z*; (ii) *x*+1/2, *y*, −*z*+1/2.
